# A mysterious sensation about sleep and health: the role of interoception

**DOI:** 10.1186/s12889-021-11603-0

**Published:** 2021-08-23

**Authors:** Teresa Arora, Mariapaola Barbato, Shaikha Al Hemeiri, Omar M. Omar, Maryam A. AlJassmi

**Affiliations:** 1 Department of Psychology, College of Natural and Health Sciences, Zayed University, Abu Dhabi, PO Box 144534, United Arab Emirates; 2 Al Jalila Children’s Specialty Hospital, Dubai, United Arab Emirates; 3 Department of Public Health, College of Health Sciences, Qatar University, Doha, Qatar

**Keywords:** Interoception, Emotional awareness, Sleep quality, Young adults

## Abstract

**Background:**

Interoception is mental awareness, recognition and acknowledgement of physiological body signals. Understanding the role of sleep and interoception may provide a better understanding surrounding the sleep-health connection. Our primary objective was to examine the potential relationships between subjective sleep quality and multiple dimensions of interoceptive abilities in a large sample of young adults, a group who are vulnerable to sleep impairment and its widespread health consequences.

**Methods:**

We conducted an online cross-sectional survey targeting young adults, aged 18–25 years. The Pittsburgh Sleep Quality Index (PSQI) was used to identify subjective sleep quality and the Multidimensional Assessment of Interoceptive Awareness Version 2 was used to assess eight domains of interoception. We conducted a series of Spearman’s bivariate correlations to assess the relationships between global sleep quality as well as the seven PSQI sub-components in relation to the eight interoception outcomes. We then conducted quantile regression to assess if global PSQI score was an independent predictor of interoception. Participants (*n* = 609) consented and provided data.

**Results:**

After adjustment, the global PSQI was a significant predictor of ‘Non-Distracting’, ‘Emotional Awareness’ and ‘Trusting’, where β = − 0.10 (95% CI: − 0.14, − 0.07), β = 0.05 (0.01, 0.09), and β = − 0.10 (− 0.14, − 0.05), respectively.

**Conclusions:**

Our findings reveal a small, significant relationship between sleep quality and interoceptive abilities amongst young adults. Sleep impairment may inhibit interoceptive skills, thus adding value to the mechanistic explanation of the sleep-health relationship. Experimental and prospective studies are needed to determine temporal associations.

**Supplementary Information:**

The online version contains supplementary material available at 10.1186/s12889-021-11603-0.

## Background

Sleep is an integral lifestyle behavior that is central to multiple physiological and psychological functions. Sleep-wake activity is regulated by the hypothalamic region of the brain and serves to maintain a range of homeostatic processes from endocrine function, internal body temperature and inflammation, to autonomic nervous system activity. A profusion of research has shown that sleep impairment can have profound effects upon diverse health outcomes from obesity [1], to dementia [2], cancer [3], and more. It is, however, important to note that the majority of research has focused on sleep duration, although some have suggested that poor sleep quality drives the onset of disease, independent of sleep duration [1]. Some have claimed that sleep is so imperative to health that, when impaired, it can contribute to the onset and progression of almost every health condition involving wide-ranging effects on cardiovascular, immune, endocrine, and nervous systems [4].

To appreciate the role of sleep pertaining to health, it is important to understand the physiological mechanisms involved. These have started to be clarified, thus enabling a deeper mechanistic understanding. For example, cross-sectional and longitudinal associations between sleep and body mass index have shown that impairments to sleep are linked to alterations in appetite-regulating hormones [5]. Research comparing ‘healthy’ sleepers to sleep-impaired individuals has consistently demonstrated that the latter group have higher subjective levels of hunger as well as elevated levels of ghrelin, a hunger-promoting hormone, thus identifying a physiological cause of weight gain [6]. Adverse metabolic effects have also been documented along with over-activation of the sympathetic nervous system in experimental sleep manipulation studies linking sleep alterations to type 2 diabetes [7]. These physiological mechanistic explanations are undoubtedly beneficial, nonetheless psychological factors are also likely to play a critical role [8]. The interplay between physiological and psychological factors are key to a better understanding. Mental awareness, recognition and acknowledgement of physiological signals, known as interoception, may enhance these mechanistic explanations and could be key to better understanding the intricate relationships between sleep and health.

The process of interoception is an interaction between the body and mind. Afferent pathways integrate internal cues and send signals to the central nervous system for evaluation. Interoception involves the processing of subtle physiological signals via neural pathways. Thus, when interoceptive abilities are functioning optimally, an individual will psychologically process, actively recognise, and become aware of physiological body states such as hunger, pain perception, the need to urinate, heart rate, emotional status, sleepiness, and more. The study of interoception is an emerging research field which has primarily focused on visceroception – physiological signals arising from organs situated within the trunk of the body. A major research focus has been specifically directed towards the cardiovascular system where objective measures include monitoring of heartbeat with participants reporting their awareness of heart rate during a pre-defined task [9]. It is, however, important to note that objective measures of interoception also require subjective reporting in order to assess discrepancies between the two measures. Given that interoception is not limited to cardiovascular output, self-reported information allows multiple aspects to be captured, thus enabling a detailed, comprehensive understanding of its role. A small amount of emerging research has suggested a link between impairment to interoceptive abilities and psychological health, particularly anxiety [10], and depression [11]. Furthermore, emerging evidence has reported sex differences pertaining to specific aspects of interoception in healthy adults, whereby women have heightened attention to internal signals and poorer interoceptive accuracy, as compared to men [12]. A recent brain imaging study revealed that this may be due to structural brain differences observed across men and women [13].

Given the well documented evidence of sleep pertaining to physiological and psychological health, it is possible that sleep may influence a range of interoceptive abilities. Moreover, given that sleep and interoception are both synchronised by neural processes, a link between the two is plausible, although not yet fully understood. Research surrounding sleep and interoception is scarce, particularly amongst non-clinical populations, although some preliminary evidence is available where healthy controls have been compared to patients with sleep or psychological disorders. One study enrolled 138 patients with past/present mental health diagnoses and compared them to 42 healthy controls [14]. Self-reported measures of sleep quality were obtained using the Pittsburgh Sleep Quality Index (PSQI) and interoception was assessed using the Porges Body Perception Questionnaire, as well as a cardiac interoception task. The findings demonstrated that poor subjective sleep quality was associated with poorer accuracy of interceptive ability amongst those with mental health conditions, particularly in those with depression [14]. A recent review highlighted a potential connection between different sleep parameters and interoception and suggested that reductions to interoceptive sensitivity during sleep may help to prevent sleep disruption. However, it is not yet known if sleep quality can predict any, or all, of the multiple dimensions of interoception. The authors concluded that a deeper understanding of these complex associations is likely to improve the management of psychological and sleep disorders, as well as patients with chronic pain and/or somatization [15].

Previous research surrounding the relationship between insomnia, a sleep disorder characterized by poor sleep quality and inadequate sleep quantity, and interoception has provided some key findings and valuable insights. One study found that those with more severe insomnia reported heightened levels of pain as well as poorer sleep quality [16]. The same study showed that after a poor night of sleep, pain reactivity was exacerbated [16]. The reverse was also veritable where a good night of sleep was shown to lower pain sensitivity [16]. In another study, an electroencephalogram (EEG) was used to determine potential differences in somatic awareness and mental activity during resting state (five-minute of eyes-closed wakefulness). Patients with chronic insomnia were compared to age and gender-matched healthy controls, with the former group having amplified somatic awareness compared to the latter [17]. One group conducted an experimental study where they manipulated core skin and body temperatures in eight older insomnia patients and compared polysomnographic-measured sleep onset latencies (SOL) to eight healthy older adults without sleep issues [18]. A small increase in skin temperature (0.4 °C) facilitated slight SOL improvements in both groups, which was also comparable to observations in healthy young adults. Interestingly, in this study, insomnia patients reported less awareness of temperature changes (blunted thermal interoception), despite the slightly improved SOL observations [18].

Based on these studies of sleep-disordered patients, it can be concluded that impaired sleep may heighten pain sensitivity as well as somatic awareness, suggesting sleep-disordered individuals may be more attentive to some interoceptive domains. However, it is less clear if poor sleep quality, as commonly experienced amongst the general population, is linked to a blunting or amplified awareness in any of the known interoceptive dimensions.

We therefore sought to examine the relationships between multiple aspects of subjective interoception and self-reported sleep quality amongst a large sample of young adults residing in the United Arab Emirates (UAE). We specifically targeted young adults from the UAE as previous research has shown that poor sleep quality [19], extreme and inconsistent sleep patterns [20], and the prevalence of psychological disorders [21] in this country, and close surrounding regions, is particularly high. Our own research also showed an exceptionally high proportion of poor sleep quality (75%), as determined by the PSQI, amongst young, female university students in one of the Emirates in the UAE [22]. Furthermore, just 22% of this sample had normal levels of anxiety and 41% had either borderline or abnormal levels of depressive symptoms [22]. Thus, the current available data suggest that the majority of emerging, young adults in one Emirate of the UAE are not only experiencing sleep difficulty but also indicate an exceptionally high vulnerability to mental ill health. This warrants further investigation and targeting of a similar population across all seven Emirates within the UAE to provide a better understanding. In turn, this will permit the design and facilitation of appropriate and targeted interventions. Moreover, it has been noted that there is an extreme shortage of facilities, resources and mental healthcare professionals to support these needs, given the country’s rapid expansion in recent decades [23]. Furthermore, youth are particularly vulnerable to the adverse effects of sleep impairment including the onset and exacerbation of mental health conditions [24]. We hypothesized that poorer quality sleep would be associated with impairments to interoceptive ability.

## Methods

Ethical approval for the study was provided by Zayed University Research Ethics Committee (ZU19_040_F) and the research adhered to the guidelines outlined in the Declaration of Helsinki. An electronic survey was developed, and our pre-defined study inclusion criteria were males and females aged 18–25 years, English language comprehension, and a UAE resident at the time of participation. Participants were not included if they had previous/current history of psychiatric or neurological conditions. Participants who consented and completed the online survey but did not meet our pre-defined study criteria were removed prior to data analysis.

### Measures

A number of demographic questions were initially asked including age (years), gender (male, female), highest education level attained (high school, college, university), nationality, and residing Emirate. These questions were developed specifically for the purposes of the present research study (see Additional file 1). The following additional measures were also employed:

### Multidimensional Assessment of Interoceptive Awareness Version 2 (MAIA-2)

This tool was developed in 2018 and was used to subjective assess interoceptive awareness [25]. The self-reported instrument contains 37 statements and response options are on a six-point Likert scale where never = 0 and always = 5. The scale is divided into eight sub-scales: 1) ‘Noticing’ which refers to the awareness of comfortable, uncomfortable and neutral body sensations and includes statements such as “When I am tense I notice where the tension is located in my body”; 2) ‘Non-Distracting’, which refers to the tendency to not ignore or distract from sensations of pain/discomfort, and includes statements such as “I ignore physical tension or discomfort until they become more severe”; 3) ‘Not worrying’ relates to a tendency to not worry or experience emotional distress with sensations of pain/discomfort and includes statements such as “I can notice an unpleasant body sensation without worrying about it”; 4) ‘Attention Regulation’ involving the ability to sustain and control attention to body sensations and includes statements such as “I can pay attention to my breath without being distracted by things happening around me”; 5) ‘Emotional Awareness’ refers to awareness of the connection between body sensations and emotional states with statements such as “I notice how my body changes when I am angry”; 6) ‘Self-regulation’ relates to one’s ability to regulate distress by attention to body sensations and includes statements such as “When I feel overwhelmed I can find a calm place inside”; 7) ‘Body Listening’ pertains to one’s ability to actively listen to the body for insight with statements such as “I listen for information from my body about my emotional state”; and 8) ‘Trusting’ relates to the experience of an individual’s body as safe and trustworthy and includes statements such as “I am at home in my body”. Scores for each sub-scale are totalled and averaged giving a range of 0–5 for each. Higher scores indicate more recognition and awareness of each of the specific interoception components. The MAIA-2 has been previously validated and has demonstrated good internal consistency reliability [25]. The instrument is freely available to use and can be downloaded for use in academic research.

### Pittsburgh Sleep Quality Index (PSQI)

The PSQI [26], is one of the most widely utilised and validated instruments for assessing sleep quality and is freely available to use for academic clinical research. It is comprised of 19 items and divided into seven components (sleep duration, sleep latency, sleep medications, daytime dysfunction, sleep disturbances, sleep quality, and sleep efficiency). The scores, which range for 0–3 (0 = best and 3 = worst), for each component are totalled giving a global PSQI score of 0–21. Higher scores indicate poorer sleep quality.

### Procedure

The survey was comprised of previously validated and reliable instruments (see previous section) and the link was shared through a range of social media sites using the snowball recruitment technique. Those who expressed an interest in study participation and clicked the link were first presented with study-related information including the eligibility criteria. Individuals were then presented with the following text under the heading ‘*What Your Consent Means’: “*Choosing the ‘Yes’ box on this consent form indicates that you have understood to your satisfaction the information regarding participation in this research project and agree to participate as a participant.” All data from participants indicating a positive consent were used in the study.

### Statistical analysis

All data were analysed using StataCorp. 2013. *Stata Statistical Software: Release 13*. College Station, TX: StataCorp LP. First, we visually inspected the distribution of continuous variables. Second, we conducted descriptive statistics for the total sample. Third, we conducted a series of Spearman’s bivariate correlations, Bonferroni corrected, to assess the relationships between each of the eight interoception sub-scales and the PSQI global score, as well as its seven individual components. Finally, we conducted four quantile regression analyses to assess if sleep quality (global PSQI score) was an independent predictor of any of the four significant interoception sub-scales, based on the Spearman’s bivariate correlation results. We developed three models as follows: 1) univariate; 2) adjusted for age and gender; 3) further adjusted for nationality, education level, and residing Emirate. Post-hoc sample size for Spearman’s correlation was determined using power analysis. The power analysis was performed using an alpha level of 5%, power of 80%, and several effect sizes (*p* = 0.15, *p* = 0.14, *p* = 0.13, and *p* = 0.12) for 2-tailed tests. On the basis of these assumptions, the estimated required sample size was determined to be *n* = 347 for *p* = 0.15, *n* = 398 for *p* = 0.14, *n* = 462 for *p* = 0.13 and *n* = 543 for *p* = 0.12.

## Results

The characteristics of the 609 participants are presented in Table 1. In brief, females represented the majority of the sample (*n* = 548; 90%). The median age was 21 years, and the highest level of education completed was college for the majority (*n* = 507; 83%). A total of 89% (*n* = 541) respondents were Emiratis and 90% (*n* = 545) of the sample were classified as poor quality sleepers based on the PSQI scoring criteria.

**Table 1:** Characteristics of 609 young adults residing in the United Arab Emirates

Characteristic	
**Sex, n (%)**	
Male	61 (10)
Female	548 (90)
**Age** (years)	21 (19–22)
**Education level**	
High school	70 (11.49)
College	507 (83.25)
University	32 (5.26)
**Nationality, n (%)**	
Emirati	541 (88.83)
Gulf/MENA (non-UAE)	46 (7.55)
Asia	13 (2.13)
Caucasian	7 (1.15)
Other	2 (0.33)
**Residing Emirate, n (%)**	
Abu Dhabi	216 (35)
Dubai	249 (41)
Sharjah	86 (14)
Fujairah	5 (1)
Umm Al Quwain	16 (3)
Ajman	23 (4)
Ras Al Khaimah	14 (2)
**Global PSQI score**	9 (7–11)
PSQI poor sleeper	545 (90)
PSQI good sleeper	58 (10)
**MAIA-2 sub-scales**	
Noticing	3.0 (2.3–3.8)
Non-Distracting	1.8 (1.0–2.5)
Not Worrying	2.2 (1.6–2.8)
Attention Regulation	2.6 (2.1–3.3)
Emotional Awareness	3.6 (2.6–4.4)
Self-regulation	2.5 (1.8–3.3)
Body Listening	2.0 (1.3–3.7)
Trusting	3.0 (2.0–4.0)

Data are presented as n (%), or median (Interquartile Range)

Caucasians was defined as those reporting nationalities from the United States of America, Europe, Australia and New Zealand

*MENA* Middle East and North Africa, *PSQI* Pittsburgh Sleep Quality Index, *MAIA-2* Multidimensional Assessment of Interoceptive Awareness Version 2

The Spearman’s bivariate correlations between global sleep quality, as well as the individual PSQI components, and each of the eight sub-scales of interoception are presented in a correlation matrix in Table 2. Global sleep quality was significantly and positively correlated with two of the eight scales: ‘Noticing’, and ‘Emotional Awareness’. It was significantly and negatively correlated with ‘Non-Distracting’ and ‘Trusting’. These four aspects of interoception were found to be significantly correlated with some other PSQI components such as sleep duration, daytime dysfunction, sleep quality and sleep disturbance.

**Table 2:** Correlation matrix to assess the relationships between global sleep quality and each PSQI component with the eight interoception sub-scales

	Noticing	Non-Distracting	Not worrying	Attention Regulation	Emotional Awareness	Self-regulation	Body Listening	Trusting
PSQI global score	0.13*	− 0.28***	− 0.01	0.00	0.14*	− 0.07	0.03	− 0.15*
Sleep duration	0.04	−0.16**	0.11	0.01	0.03	−0.04	− 0.06	− 0.08
Sleep latency	0.06	−0.12	− 0.01	− 0.01	0.08	− 0.10	−0.00	− 0.07
Daytime Dysfunction	0.14*	−0.26***	−0.05	− 0.04	0.15**	− 0.05	0.04	− 0.16**
Sleep efficiency	−0.02	− 0.07	0.04	− 0.01	−0.01	− 0.01	−0.04	− 0.07
Sleep quality	0.13*	−0.24***	−0.06	0.04	0.07	−0.05	0.05	−0.11
Sleep medication	0.04	−0.08	0.01	0.03	0.05	−0.04	−0.01	− 0.07
Sleep disturbance	0.12	−0.14*	−0.10	− 0.00	0.17***	0.03	0.09	−0.09

*p < 0.05; **p < 0.01; ***p < 0.001 (Bonferroni corrected)

The results from the quantile regression analysis are highlighted in Table 3. For those in the 50th percentile, each one-unit increase in the PSQI global score was associated with a 0.03 significant increase in the interoception sub-scale of ‘Noticing’, after adjustment. The strongest effect size, after adjustment, was observed for ‘Trusting’ and ‘Non-Distracting’, where the corresponding beta coefficients (95% CIs) were − 0.10 (− 0.14, − 0.05) and − 0.10 (− 0.14, − 0.07), respectively. The sub-scale ‘emotional awareness’ was also significant after adjustment, where β = 0.05 (0.01, 0.09).

**Table 3:** Quantile regression for sleep quality as an independent predictor of four interoception sub-scales

	***Model 1***	***Model 2***	***Model 3***
**Noticing**			
50th percentile	0.04 (− 0.00, 0.07)	0.03 (− 0.00, 0.07)	0.03 (− 0.00, 0.07)
**Non-Distracting**			
50th percentile	−0.10 (− 0.14, − 0.06)***	−0.10 (− 0.13, − 0.06)***	−0.10 (− 0.14, − 0.07)***
**Emotional Awareness**			
50th percentile	0.05 (0.01, 0.09)*	0.05 (0.01, 0.10)*	0.05 (0.01, 0.09)*
**Trusting**			
50th percentile	−0.10 (−0.15, −0.06)***	− 0.10 (− 0.14, − 0.05)***	−0.10 (− 0.14, − 0.05)***

Data are presented as β coefficient (95% Confidence Intervals)

Model 1: unadjusted

Model 2: adjusted for age and sexo

Model 3: further adjusted for nationality, Emirate, and education level

*p < 0.05; ***p < 0.001

## Discussion

We investigated the possible relationships between subjective sleep quality and multiple dimensions of interoception in a large sample of young adults. Of particular note was the high proportion of participants who met the PSQI scoring criteria for poor sleep quality (90%). We further observed consistent, significant correlations between various sleep parameters and four features of interoception, namely: ‘Noticing’, ‘Non-Distracting’, ‘Emotional Awareness’ and ‘Trusting’. Our regression analyses confirmed small but statistically significant associations between the latter three interoceptive measures. Our study findings suggest that sleep is likely to play a small but vital role in understanding interoceptive abilities amongst young adults. Importantly, this age group are vulnerable to the effects of sleep impairment [27], which is known to contribute to a heightened risk of mood deficits as well as psychological disorders [24].

As with any physical and psychological function, homeostasis is paramount for ensuring the body remains in equilibrium. Disruptions to sleep homeostasis, whether that be sleep duration, sleep quality or sleep-wake timings, have been associated with a multitude of health conditions. Our novel results also suggest that poorer sleep quality may be associated with interoceptive sensibility. Whilst this is a limited research area, particularly amongst non-clinical populations, our observations make intuitive sense. Those with impaired interoceptive abilities may be deficient in recognising physiological cues or, mechanistically, psychological and cognitive processing ability may be diminished. Similarly, studies have shown that experimental sleep manipulation results in poorer cognitive outcomes [28]. So, if poor sleep quality drives interoceptive imbalance, as previous research has suggested [14], then this may be explained by cognitive blunting, resulting in diminished recognition of internal bodily changes. Alternatively, those with poor sleep quality may also have heightened interoceptive awareness through over-activation of the sympathetic nervous system [29], which would explain previous findings pertaining to cardiac interoception and sleep quality [14]. If, on the other hand, poor sleep quality is a consequence of impaired interoceptive abilities, then this could potentially be driven by neurological factors, given that the brain regulates sleep behaviour. Impairments to interoceptive sensibility have been shown to predict an increased risk of psychosis [30]. Thus, as affective psychopathology is closely connected to sleep disruption [14], impairments to interoceptive abilities may elicit psychological and neurological alterations with subsequent changes to mood and sleep behaviour.

Disregarding physiological signals of sleepiness is widespread, which has been linked to multiple adverse outcomes in young populations, including an increased likelihood of road traffic accidents, substance use, as well as behavioural and health conditions [31]. Conversely, selective attention to physiological signals such as increased heart rate, as a consequence of hyper-interoceptive awareness, may contribute to the onset or exacerbation of conditions, such as anxiety and insomnia [32]. Furthermore, heightened awareness of hunger signals which are driven by sleep loss through the effect of altered hormones [6], may help to further explain the relationship which has been consistently observed between sleep and obesity [1, 33], as well as type 2 diabetes [34]. It is possible that interoception potentially mediates the pathways between sleep and psychological and physiological health conditions. This notion is, however, still to be confirmed through a series of carefully designed research studies. Given that we collected cross-sectional data, we cannot be certain of directional associations. For example, it is possible that poor sleep quality could impair interoceptive abilities, but the reverse is also possible, whereby impairments to interoceptive skills could result in poorer sleep habits, quality or duration. The precise directionality needs to be confirmed through a series of experimental and prospective studies that involve a wider range of targeted populations.

Our findings revealed that global sleep quality was significantly associated with three specific dimensions of subjective interoceptive experience, after adjustment. These specific aspects are interesting in relation to sleep and require further discussion. For example, ‘Non-Distracting’ and ‘Trusting’ both had the strongest effect sizes and were negatively associated with sleep quality. The interoceptive measure of ‘Non-Distracting’ refers to an individual’s tendency to not ignore or distract away from sensations of pain/discomfort. From an interpretative viewpoint, our observations suggest that individuals who tend to acknowledge such sensations have better sleep quality (a lower global PSQI score). This implies that compared to individuals with poor sleep quality, those with good sleep quality might be more likely to recognise and possibly take action to deal with uncomfortable body sensations such as pain and hunger. Moreover, our results also demonstrate that individuals with better sleep quality are also more trusting of their body and its signals. This is consistent with previous research in the area of sleep and subjective hunger [6], as well as pain tolerance [35]. For example, at least one study has confirmed that poorer sleep quality (measured using the PSQI) is significantly associated with higher levels of perceived hunger as well as greater disinhibited feeding behaviour [36]. Other research, which compared healthy controls to women with Endometriosis, showed that the latter group had poorer subjective sleep quality. Interestingly, however, no group differences were observed surrounding pain threshold and sleep quality, which were positively correlated [37].

The concept of ‘Emotional Awareness’ as an interoceptive dimension also requires further discussion. Our findings showed a positive association between this aspect and sleep dysfunction, meaning that those with greater awareness of the connections between body sensations and emotional states tend to score higher on the PSQI, indicating poorer sleep quality. There is an overwhelming amount of evidence suggesting the role of sleep pertaining to emotion regulation. One recent study of young, low-income women from diverse ethnic backgrounds, showed that negative mood was associated with poor sleep quality through two specific pathways in path analysis. First, the two variables were directly and significantly associated. Second, an indirect link was observed between negative affect and sleep quality through the mediated effects of difficulties with emotional regulation [38]. Moreover, a recent mechanistic study examined objective and subjective sleep measures along with an emotion regulation task involving reappraisal [39]. The authors concluded that disturbances to sleep is likely to play a role in emotion dysregulation in those with anxiety and/or depressive disorders. Interestingly, subjective and objective sleep measures were uncorrelated, and the authors suggested that this clinical population may have difficulties with cognitive reappraisal skills [39]. We believe that this could be driven by deficits to interoceptive abilities. Heart rate variability (HRV) is a well-known psychophysiological indicator of physiological arousal and emotion regulation which can predict stress and sleep disturbance, both of which are associated with greater odds of depressive symptoms. In one study, poor sleep quality mediated the relationship between stress and depressive symptoms leading the authors to conclude that a lower HRV poses greater susceptibility to stress-driven sleep impairments, which then heightens the risk for depressive symptoms, as a response to chronic stress [40]. Whilst these findings unequivocally aid our understanding of the associations between sleep and emotion regulation, we propose that interoception could be a missing piece of the mind-body interaction puzzle, which may further inform mechanistic explanations.

Given that sleep behaviour and interoceptive processes are coordinated by the brain, structural and functional brain imaging studies have provided some vital clues about the relationship between these two aspects. It has been suggested that specific brain structures which regulate the response to environmental stimuli and subsequently processed by the central nervous system may, in part, explain the interoceptive findings observed in those with insomnia [41], as well as sex differences [12, 13]. Furthermore, a case-control study, which compared 24 chronic insomnia patients to 13 matched healthy controls, found lower grey matter volume within the orbitofrontal cortex amongst the sleep-disordered group compared to healthy controls [42]. Another comprehensive brain imaging study confirmed that lower grey matter volume in the orbitofrontal region was associated with early morning awakenings in insomnia patients, a common symptom of this condition [43]. What is not currently known, however, is if the lower volume of grey matter in those with insomnia is a cause or consequence of persistent sleep problems which warrants further prospective, comprehensive investigation.

Interestingly, biofeedback equipment is sometimes used as part of the therapeutic process in patients to raise awareness of internal physiological processes and body signals [44]. Recognition of these internal cues enhances interoceptive skills by helping patients to improve their control over the symptoms of anxiety (increased heart rate, hyperventilation, body temperature) through the effects of visual physiological feedback. Equally, it can be used to demonstrate to patients the physiological effects upon the parasympathetic nervous system following progressive muscle relaxation, controlled breathing techniques and/or mindfulness-based stress reduction programmes, thus enhancing interoceptive accuracy pertaining to non-contingent cardiac feedback [45]. We recommend, however, a combination of sleep improvement as well as suitable adjustments to interoceptive skills to potentially combat psychological disorders and prevent incident cases in at risk populations. Recent evidence has demonstrated that cognitive behavioural therapy for insomnia (CBT-I) exhibits positive sleep outcomes and can also help to alleviate depressive symptoms [46]. However, it should be noted that biofeedback was not explicitly described as part of the intervention, thus it remains unknown as to if it was incorporated.

Our study is the first to examine the associations between sleep quality and multiple subjective dimensions of interoception. Other strengths of our study include the specifically targeted sample of young adults who are both vulnerable to the effects of sleep loss and at higher risk of developing mental health difficulties. Moreover, the sample size for an emerging and novel area of research was relatively large. We also acknowledge a number of study limitations. First, our study was cross-sectional, thus we cannot draw any conclusions about temporal associations. For example, it is possible that poor sleep habits may impair interoceptive capabilities. An equally feasible explanation is also that poorer interoception could hinder sleep outcomes, thus directionality is still to be confirmed. Second, our sample was comprised of young adults residing in the UAE and the majority of respondents were female, thus our findings may not be generalizable to other age groups, males, or those in other countries and we recommend study replication amongst these populations to confirm or refute the findings. Furthermore, it was not possible to examine sex differences due to the predominance of females in our sample. Future studies could explore possible sex differences given that notable differences in interoception [12, 47] and sleep [48], as well as brain structures [13] have been previously documented. Third, all data acquired were subjective, however, we were certain to include previous validated instruments which had been assessed for reliability pertaining to sleep quality and interoception to enhance the quality of data. That said, we recognise that there may be some biases such as recall, and social desirability. We also acknowledge the potential of response bias with those experiencing sleep problems perhaps being more likely to complete the survey, given that 90% of our sample met the criteria for poor sleep quality based on accepted PSQI cut points. Although published data on sleep is scarce amongst non-clinical populations in the UAE, we propose that our data is reasonably representative of the UAE student population. Data from surrounding geographical regions has shown a high prevalence (60%) of poor sleep quality [19]. Other groups have revealed extreme and inconsistent sleep patterns amongst students from Saudi Arabia, with a 65% prevalence of sleep disturbance reported in this group [20]. Finally, Arabic is the national language and our online survey was provided in English only. However, English is widely spoken in the UAE, particularly amongst the younger generations, but we acknowledge that some participants may have misinterpreted the meaning of some questions. This, in turn, may have affected some the responses provided. Moreover, given that the survey was made available in English only, this may have attracted a more educated sample, thus our findings may not be a fully accurate representation of young adults residing within the UAE.

## Conclusions

The proportion of respondents meeting the criteria for poor sleep quality was excessively high. Given the limited literature surrounding sleep in this geographic region amongst young people, our findings undoubtedly warrant further investigation of more detailed sleep habits amongst youth across Middle Eastern prosperous countries. Despite the effect sizes observed in our regression analyses being small, our findings indicate a novel and significant connection between subjective sleep and multiple aspects of interoception. We recommend that acute, experimental sleep lab-based studies are conducted to determine cause-effect relationships and that these should also be paired with prospective, longitudinal studies to investigate if sleep impairment, or disruptions to sleep architecture, causes alterations to interoceptive abilities, whilst also considering sex differences. We also suggest that interoceptive skills may mediate the relationship between sleep and psychological disorders although this also needs to be confirmed or refuted in further research studies. Given the scarcity of the literature surrounding interoception and sleep, more research is needed in order to provide a better understanding of the intricate relationships. That said, we believe that interoception is instrumental to the mechanistic explanation which is likely to underpin the association between sleep and health. For example, if poor sleep does impair interoceptive abilities, such as hunger and satiety, then the downstream consequences of this may adversely affect dietary habits and subsequent onset or exacerbation of diet-driven chronic diseases such as obesity and type 2 diabetes mellitus. Achieving optimal and balanced interoceptive skills through interventional studies may be key to ensuring better health and wellbeing, despite the current infancy of this research field.

## Supplementary Information

**Additional file 1.**

## Data Availability

The dataset used and analysed during the current study are available from the corresponding author on reasonable request.

## Abbreviations

CBT-I: Cognitive Behavioural Therapy for Insomnia
EEG: Electroencephalogram
MAIA-2: Multidimensional Assessment of Interoceptive Awareness Version 2
PSQI: Pittsburgh Sleep Quality Index
SOL: Sleep Onset Latency
UAE: United Arab Emirates
