# Supplementary material for: A mysterious sensation about sleep and health: the role of interoception
Source: BMC Public Health. 2021 Aug 23;21:1584. doi: 10.1186/s12889-021-11603-0 (PMC8381551; doi:10.1186/s12889-021-11603-0)
Supplement: Supplementary file 1 — Additional file 1. [file 12889_2021_11603_MOESM1_ESM.docx]

**Demographic questions**

1. How old are you? (only age 18-25 can participate): ______years
2. What is your gender? Male Female

4) What is the highest level of education you have completed?

1. Did not attend school
2. 1st grade
3. 2nd grade
4. 3rd grade
5. 4th grade
6. 5th grade
7. 6th grade
8. 7th grade
9. 8th grade
10. 9th grade
11. 10th grade
12. 11th grade
13. Graduated from high school
14. 1 year of college
15. 2 years of college
16. 3 years of college
17. Graduated from college
18. Some graduate school
19. Completed graduate school
20. What is your nationality?
21. UAE
22. Gulf (non-UAE)
23. Asia
24. America
25. Europe
26. Australia/New Zealand
27. Other ___________
28. In which Emirate do you live?
29. Abu Dhabi
30. Dubai
31. Sharjah
32. Fujairah
33. Umm Al Quwain
34. Ajman
35. Ras Al Khaimah
